# Bile Cast Nephropathy: A Comprehensive Review

**DOI:** 10.7759/cureus.23606

**Published:** 2022-03-29

**Authors:** Manoj R Somagutta, Molly S Jain, Maria Kezia Lourdes Pormento, Siva K Pendyala, Narayana Reddy Bathula, Nagendrababu Jarapala, Ashwini Mahadevaiah, Nayana Sasidharan, Mohamed A Gad, Greta Mahmutaj, Namrata Hange

**Affiliations:** 1 Department of Medicine and Research, Avalon University School of Medicine, Willemstad, CUW; 2 Department of Research, California Institute of Behavioral Neurosciences & Psychology, Fairfield, USA; 3 Medicine, Saint James School of Medicine, Park Ridge, USA; 4 Medicine, Ateneo de Manila School of Medicine and Public Health, Quezon City, PHL; 5 Department of Medicine, Atlantic University School of Medicine, Gross Islet, LCA; 6 Department of Medicine, Jagadguru Sri Shivarathreeshwara (JSS) University, Mysore, IND; 7 Department of Medicine, Academy of Medical Sciences, Trivandrum, IND; 8 Department of Family and Community Medicine, Saint George's School of Medicine, St. George's, GRD; 9 Department of Medicine, The University of Medicine, Tirana, Tirana, ALB; 10 Public Health, Eurasian Cancer Research Council, Mumbai, IND

**Keywords:** acute kidney injury, bile acid, bilirubin, cholemic nephropathy, bile cast nephropathy and renal failure

## Abstract

Bile cast nephropathy (BCN) or cholemic nephropathy (CN) is an acute renal dysfunction, including acute kidney injury (AKI) in the setting of liver injury. It is a common phenomenon in patients with liver disease and is associated with significant morbidity and mortality. CN is characterized by hemodynamic changes in the liver, kidney, systemic circulation, intratubular cast formation, and tubular epithelial cell injury. CN has been overlooked as a differential diagnosis in chronic liver disease patients due to more importance to hepatic injury. However, frequent and considerable reporting of case reports recently has further investigated this topic in the last two decades. This review determines the evidence behind the potential role of bile acids and bilirubin in acute renal dysfunction in liver injury, summarizing the implied pathophysiology risk factors, and incorporating the therapeutic mechanisms and outcomes.

## Introduction and background

The term bile cast nephropathy (BCN), also known as cholemic nephropathy, icteric nephrosis, or cholemic nephrosis, is described as acute renal dysfunction, including acute kidney injury (AKI) in the setting of liver injury [1]. Quincke first reported it in 1899 during autopsy examinations of patients with acute jaundice and renal insufficiency. BCN is a multidimensional entity resulting in tubular and interstitial inflammation, tubular obstruction, direct bile salt-induced tubular toxicity, and altered renal hemodynamics [2,3]. The attribution of AKI to bile acids and bilirubin is debatable due to bilirubin’s protective effects [1,4]. This topic was actively discussed in the early 1900s but somehow not well investigated, contributing to its limited appearance in the current medical literature [1,4]. The probable reasons it was overlooked may be because of a lack of consensus in the mechanism of the CN and diagnostic modalities in confirming the diagnosis [1,3].

The AKI in cholestatic liver dysfunction is usually linked with other unfavorable factors such as hypovolemia, endotoxemia, and exposure to nephrotoxins [5]. On the other hand, the AKI in chronic liver injury patients is frequently attributed to hepatorenal syndrome (HRS), which is characterized by alternating intrarenal vasoconstriction and splanchnic vasodilation leading to functional and hemodynamic changes in the kidney [2,5,6]. HRS could be described as type 1 and type 2. Type 1 HRS is a rapid renal failure with a serum creatinine level rising greater than 2.5 mg/dL in less than two weeks and is known for causing AKI. On the contrary, type 2 HRS is defined as a slower moderate decline in renal function with serum creatinine levels ranging between 1.5 and 2.5 mg/dL resulting in refractory ascites [7]. The definitive diagnosis of BCN is made by renal biopsy. However, the presence of impaired coagulation profile in most liver injury patients at the time of presentation makes kidney biopsy almost impracticable to perform, thus posing a diagnostic challenge [1,2,6]. For all these reasons, BCN is frequently overlooked as a differential diagnosis of AKI in obstructive jaundice patients. However, van Slambrouck et al. suggest a notable overlap between the two entities as the reason for AKI [3]. Moreover, several case reports have been reported where patients with suspected BCN treated presumptively have shown striking outcomes. Further, frequent and considerable reporting of case reports in the last two decades has yielded the need for further investigation in this topic.

BCN is underdiagnosed and is witnessed as a neglected injury found in the autopsy of patients with hyperbilirubinemia and renal dysfunction. The pathophysiology of AKI in the setting of hyperbilirubinemia is multifactorial and involves a wide range of mechanisms. This review aims to determine the evidence behind the potential role of bile acids and bilirubin in acute renal dysfunction in liver injury. It also summarizes the implied pathophysiology risk factors and incorporates the therapeutic mechanisms and outcomes.

## Review

Bilirubin and cholemic nephropathy

Excess bilirubin has detrimental effects on kidney tubule function and intracellular mitochondrial function. Bilirubin aids in oxidative stress to kidney tubular epithelium leading to damage to the tubules and associated renal structures [2,4,8]. A significant finding for this damage is tubular hypertrophy, as seen in 73.5% of autopsies of jaundiced patients [4]. Additionally, more histological findings noticed that bilirubin’s disastrous effects included tubular epithelium swelling, hypertrophy, and hyperplasia of the parietal layer of Bowman’s capsule and the formation of pigmented casts [2,4]. These kidney architectural changes are irreversible in pathology, forming fibrosis in the kidney interstitial tissue, leading to tubular atrophy [4,8].

**Figure 1:**
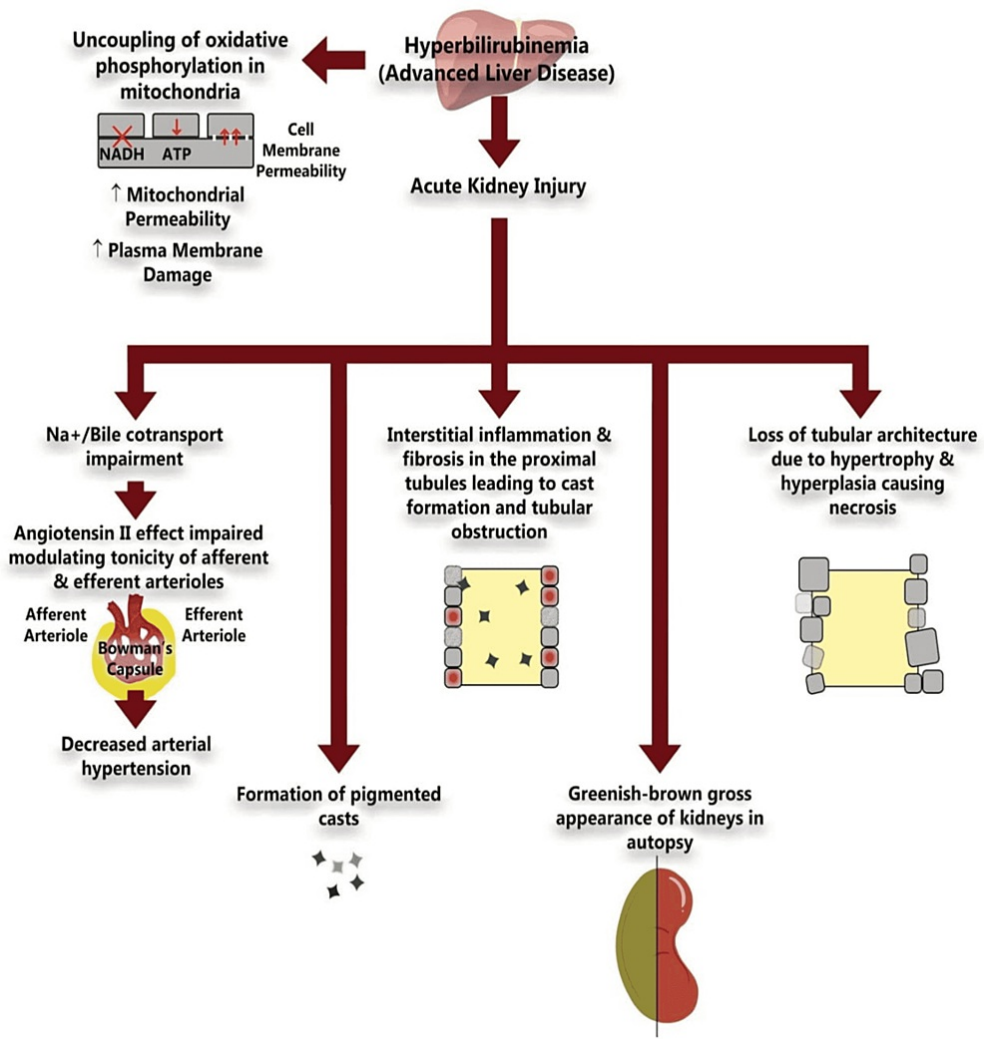
Summary of the Pathophysiological Mechanism of Cholemic Nephropathy Original image created by the authors. NADH: nicotinamide adenine dinucleotide; ATP: adenosine triphosphate

Moreover, hyperbilirubinemia can cause uncoupling oxidative phosphorylation in the mitochondria [2,8]. Oxidative phosphorylation is significant in forming adenosine triphosphate (ATP), the central energy-producing molecule in human cells. This decrease in ATP contributes to electrolyte imbalance and cell membrane penetration with increased cell volume leading to significant reversible and irreversible changes in the mitochondria [4,8]. Additionally, inhibition of Na-H, Na-K, Na-Cl pumps due to bile salts can result in cast formation, causing pH alterations in the proximal tubule and loop of Henle, leading to tubular toxicity [3,4]. Hyperbilirubinemia decreases angiotensin II-mediated arterial hypertension by reducing the production of superoxide and sodium reabsorption in the thick ascending loop of Henle [9]. Moreover, AKI induced by elevated bilirubin has been evidenced histologically with loss of expression of aquaporin-2 channels in collecting ducts in the patients diagnosed with CN [10]. The potential mechanism of CN is illustrated in Figure 1.

In contrast to bilirubin’s harmful effects, it also exhibits debatable renoprotective effects [1]. It is attributed to the lack of heme and iron, which contribute to renal injury [4]. Moreover, several animal studies have shown an increase in the enzyme heme oxygenase due to common bile duct ligation, which has anti-inflammatory, anti-apoptotic, and anti-oxidant effects exhibiting even more cytoprotective effects [1]. Hence, it is questionable if bilirubin is entirely harmful to kidneys, and more research is needed to investigate further.

Bile acids and cholemic nephropathy

Most bile acids are usually reabsorbed in the ileum and transported via portal blood circulation back to the liver. It is estimated that the liver does not take up about 10-50% of reabsorbed bile acids, making them escape in the peripheral circulation leaving behind 100 μmol bile acids per day prone to glomerular filtration [1,6]. Organic solute transporters such as apical sodium-dependent bile acid transporter (ASBT) in proximal tubules control the renal-hepatic circulation reabsorbing the filtered bile acids [1]. Under normal physiologic conditions, approximately 1-2 μmol per day of bile acids are excreted in urine [1].

With the above theory in context, it is worth noting how these mechanisms are affected in the event of cholestatic diseases such as obstructive jaundice, primary biliary cirrhosis, and primary sclerosing cholangitis. To counteract the rising bile salts in these pathologies, hepatocytes heighten basolateral hepatocellular export and enhance renal filtration and tubular secretion in the proximal tubules via organic anion transporters and multidrug resistance-associated proteins [1,6]. There seems to be an imbalance between tubular reabsorption and tubular secretion, with excessive bile salts getting excreted, thereby exceeding the maximum capacity of proximal tubules contributing to renal injury [4,6]. In Krones et al.’s study, they noticed that long-term common bile duct ligation (CBDL) in a mice model led to cholemic nephropathy because of severe cholestasis and renal instead of biliary secretion of bile acids leading to tubular epithelial injury, cast formation, basement membrane defects, and ultimately kidney fibrosis [11]. In another study by Fickert et al., excess urinary elimination of bile acids was potentially toxic to the renal tubular epithelial cells, causing kidney injury, which affected the region of aquaporin-2-collecting ducts, further strengthening the detrimental effects of bile acids on renal physiology [12]. Bile acids also directly mediate the formation of vasoactive mediators that cause renal vasoconstriction and a decrease in glomerular filtration rate (GFR) [1,4].

Hemodynamic changes due to hyperbilirubinemia

HRS is a life-threatening complication that compromises renal function, especially in patients with advanced liver disease [7-10]. The underlying mechanism is not entirely understood but is attributed to the changes in physiological aspects of HRS. Significant events in advanced cirrhosis such as severe portal hypertension, splanchnic vasodilation and arterial underfilling, and vasoconstrictor activation (renin-angiotensin-aldosterone, endothelin) lead to renal vasoconstriction and hypoperfusion, eventually causing AKI [4,13]. Studies performed on mice have demonstrated adverse chronotropic and inotropic effects on the heart due to hyperbilirubinemia leading to renal hypoperfusion [4]. Furthermore, treating hyperbilirubinemia has been shown to improve “jaundiced heart,” a common term reflecting a decline in cardiac performance in advanced hepatic dysfunction [4]. The evidence suggests that systemic endotoxins release due to excess bilirubin, leading to renal blood flow redistribution, causes hypotension and hypoperfusion. It eventually leads to corticomedullary junction ischemia and results in tubular injury [1,4]. A similar mechanism is linked with concomitant sepsis-related-AKI in these patients [4].

Moreover, in patients with liver cirrhosis, endotoxins are released due to the translocation of bacteria and pathogen-associated molecular patterns (PAMPs) from the gut and prompt systemic inflammation [1,4]. Specifically, toll-like receptor 4 (TLR4) was observed to be increased in the urine of patients with liver dysfunction, AKI, and inflammatory insults [1,4]. Altogether, these factors contribute to lowering the GFR and causing AKI. We summarized the available evidence supportive of CN in Table 1.

**Table 1:** Summary of clinical features seen in case reports of cholemic nephropathy *computed from mmol/L to mg/dL; (T): total bilirubin; (D): direct bilirubin; EBV: Epstein–Barr virus; ATN: acute tubular necrosis; Cr: creatinine; s/p: status post; TCF2: transcription factor 2; ECAD: extracorporeal albumin dialysis; MARS: molecular adsorbents recirculation system; SPAD: single-pass albumin dialysis; CBD: common bile duct; ERCP: endoscopic retrograde cholangiopancreatography; Md: median

Author, year	Subjects	Cause	Peak bilirubin level (mg/dL)	Cr level (mg/dL)	Biopsy Findings	Therapy	Outcome
Torrealba et al., 2018 [13]	2	Patient A: alcoholic steatohepatitis. Patient B: Wilson’s disease	Patient A: 37.82* (T) 20.01* (D). Patient B: 43.24* (T) 31.08* (D)	Patient A: 2* Patient B: 2.5*	Granular, pigmented tubular casts with red coloration and green-brown pigment within tubular epithelial cells, severe tubular necrosis, bile casts with positive green staining	-	Patients expired
Kiewe et al., 2004 [14]	1	Hodgkin’s lymphoma	30.4 (D)	1.7	Hypertrophy of tubular epithelium bile casts in distal and collecting tubules	Hemodialysis	Resolution of kidney injury and discontinuation of hemodialysis
Betjes and Bajema, 2006 [15]	2	Obstructive jaundice in patient A, autoimmune hepatitis in patient B	36.4 (T) 33.2 (T)	-	Bilirubin pigment in the tubules. Tubular cell necrosis	-	Improvement of renal function along with decrease in bilirubin level in patient A. Patient B died
Uslu et al., 2010 [16]	20	Obstructive jaundice	10.1 (T)	-	Dilatation of peritubular venules, acute tubular necrosis	-	Absolute recovery of renal function in all patients after biliary drainage
Bredewold et al., 2011 [17]	1	EBV infection	28.5 (D)	3.25	ATN features abundant bile casts	Hemodialysis	Resolution of infection and hyperbilirubinemia. Discontinuation of hemodialysis
Rafat et al., 2013 [18]	1	Cholangiocarcinoma	20.9 (D)	6	Presence of tubular damage: loss of brush border, tubular necrosis. Bile casts and thrombi in proximal and distal tubules	Renal replacement therapy	Patient passed away
Van Slambrouck et al., 2013 [3]	24	Obstructive cholestasis	24.9 (T)	-	Bile casts with involvement of distal nephron segments	-	-
Luciano et al., 2014 [19]	1	Anabolic steroid abuse	7.9 (T)	2.9	Multiple green-brown casts in the distal tubules. Diffuse ATN with dilatation of tubular lumen, vacuolization of tubular cell cytoplasm, and apical blebbing	No hemodialysis	Kidney function improved over 4 months and Cr plateaued at 1.8 mg/dL
Van der Wijngaart et al., 2014 [20]	1	Obstructive jaundice with multiple gallstones in the common bile duct	39.6 (T)	7.35	Bile casts, reactive changes of tubular epithelial cells	Hemodialysis, biliary drain	Improvement of kidney function after 5 weeks
Jain et al., 2015 [21]	1	Colorectal cancer s/p wedge resection of liver	42.5 (T) 25 (D)	2.72	Intratubular bile casts	-	-
Tabatabaee et al., 2015 [22]	2	Stanozolol abuse	28 (D)	8.7	Preserved glomeruli. Degeneration of cortical tubules. Bile casts present in some tubules	Hemodialysis	Cr level decreased at 2 months
Alkhunaizi et al., 2016 [23]	1	Anabolic steroid abuse	29.9 (D)	2.6	Glomeruli were unremarkable. Acute tubular injury with luminal ectasia. Dark green bile casts within tubular lamina	Supportive care only, no hemodialysis	Serum Cr returned to normal and serum total bilirubin dropped to 1.8 mg/dL at 3-month follow-up
Sequeira and Gu, 2015 [24]	1	Acute alcoholic hepatitis	20 (D)	9.2	Normal glomeruli intratubular bile casts shown by Hall’s Stain	Hemodialysis	Urine output improved gradually, however, the patient continued to need dialysis for poor clearance
Alalawi et al., 2015 [25]	1	Acute liver injury	7 (T)	7.3	Positive Fouchet stain indicating presence of bilirubin casts	Seven sessions of hemodialysis	Recovered kidney function. Discharge Cr = 1.1 mg
Flores et al., 2016 [26]	1	Anabolic steroid-induced cholestasis	53 (T)	2.3	Yellow, brown intraluminal tubular casts. Flattening and simplification of the epithelial lining	Five sessions of plasmapheresis, no hemodialysis	Bilirubin level decreased Cr level decreased and the patient recovered kidney function
Alnasrallah et al., 2016 [5]	1	Flucloxacillin-induced liver injury	34 (D)	6.6	Normal glomeruli. Positive bile stain and bile casts in tubules	No hemodialysis	Bilirubin level decreased Cr level decreased to stabilize at 1.85 mg/dL
Sens et al., 2016 [27]	1	TCF2 mutation-induced biliary duct dystrophy	15.2 (D)	5.8	Acute tubular injury: dilated tubules with flattened epithelium greenish-brown intraluminal casts	Hemodialysis 9 ECAD:1 MARS and 8 SPAD sessions	The patient underwent simultaneous liver-kidney transplant
Mukherjee et al., 2019 [28]	1	Severe hepatic dysfunction	41.7 (T) 23.4 (D)	8.2	Bile casts in renal tubules on Hall’s stain with bile staining of necrosed cells and tubular casts	No hemodialysis	Patient died
Patel et al., 2016 [8]	1	Acute liver injury	29 (T)	5.47	Proximal and distal tubules containing bile casts	Hemodialysis	The patient underwent simultaneous liver and kidney transplants. Normalization of kidney and hepatic indices
Werner et al., 2016 [29]	1	Painless jaundice due to cholangiocellular carcinoma	-	-	Dilated tubules, bile casts	Hemodialysis, bile duct stent	Resolution of renal function after restoration of cholestasis
Mohapatra et al., 2016 [30]	20	Severe falciparum malaria complicated with jaundice	26.5 (T)	-	Numerous tubular casts, acute tubular necrosis but maintained glomerular architecture	-	Recovery time of renal dysfunction 15.1 ± 6.5 days
Leclerc et al., 2016 [31]	1	Drug-induced hepatic jaundice	30.93 (T)	7.1	Brown casts clog the tubular lumen, brown deposits in the cytoplasm of tubular epithelial cells	Hemodialysis	Improvement of kidney function after normalization of bilirubin and hemodialysis
Aniort et al., 2017 [32]	1	CBD stones induced obstructive jaundice	32.6 (T)	5.3	Bilirubin tubular casts predominated in distal tubules	ERCP, cholecystectomy	Kidney function fully recovered to Cr level of 0.9 mg/dL after 3 months
Jung, 2017 [33]	1	Acute hepatitis A	10.29 (T) 7.95 (D)	14.3	Renal tubular lumen contained dark pigmented casts with foreign body reactions and calcifications, and interstitium focally exhibited mononuclear cell infiltration and fibrosis	-	-
El Khoury et al., 2017 [34]	1	Anabolic steroids	37.9 (T) 32.1 (D)	2.2	The patient refused renal biopsy	Six sessions of plasma exchange	Asymptomatic after 3 months
Sood et al., 2017 [35]	1	Acute liver failure	30.9 (T)	-	Bile cast and tubular epithelial injury in the form of lowering of epithelium, vacuolization, and necrotic debris in the lumen	-	-
Foshat et al., 2017 [36]	55	Hepatitis C virus infection (52%)	10.4 +/- 12.0 (mean +/- SD)	2.8 +/- 2.1	At least one intratubular Hall-positive cast	Nine patients had continuous venous-venous hemofiltration dialysis or hemodialysis	-
Nayak et al., 2017 [37]	57	Decompensated cirrhosis and acute on chronic liver failure	Md (range): 27.0 (1.5-72.8) 16.3 (0.2-45.8) (D)	2.6 (1.5-10.3)	Bile casts were positive according to green color on Fouchet’s staining and negative Perl’s stain in at least one tubular lumen	-	-
Pitlick and Rastogi, 2017 [2]	2	Patient A: alcoholic hepatitis. Patient B: drug-induced liver injury secondary to Augmentin	Patient A: 35.3 (T) Patient B: 37.6 (T)	Patient A: 11.1 Patient B: 3.2	ATN and casts consistent with bile	No hemodialysis	Patient A: Cr decreased, and urine output began to rise on hospital day 28. Patient B: after 2 months, Cr was 1.4 and bilirubin was 1.1
Van de Ven et al., 2018 [38]	1	Obstructive jaundice	24.9* (T) (estimated from graph)	5.42*	Refrained from kidney biopsy	Five sessions of hemodialysis	Renal function improved to normal within 3 months
Chan et al., 2019 [9]	1	CBD stones induced obstructive jaundice	32.18* (T)	5.23*	Many tubules contained yellow to green casts, some of which were birefringent to polarized light	ERCP with sphincterotomy and stent insertion	Recovered after 3 months
Ravi et al., 2018 [39]	1	Acute hepatitis A	40 (T)	11	Normal glomeruli and interstitial edema with tubules containing pigmented casts	Hemodialysis	The patient’s renal function started to improve 6 weeks after dialysis
Fisler et al., 2018 [40]	1	Acute liver injury	39.78* (T)	3.5*	Acute kidney injury with dilatation and necrosis of the renal tubules as well as intraluminal pigmented casts	Hemodialysis	-
Bräsen et al., 2019 [10]	8	Viral cause of liver disease (37.5%)	Max: 45.57 +/- 17.9*	4.7 +/- 3.5*	-	-	-
Giuliani et al., 2020 [41]	1	CBD stones induced obstructive jaundice	28.08* (T)	4.8*	Many tubules contained brown casts	-	-
Jamshaid et al., 2020 [42]	1 (64 M)	Obstructive jaundice	30.01* (T) 23.93* (D)	5.6*	Not performed	Hemodialysis	Improved after 3 sessions of hemodialysis

Approach and diagnosis

Currently, a kidney biopsy is the gold standard diagnostic test for CN. In autopsy evaluations of CN patients, the kidney’s cortex and medulla appear yellow due to bilirubin. After fixation with formalin, color changes to green due to bilirubin oxidation and conversion to biliverdin [1,27]. On histological diagnosis, yellow or green-brown casts will be seen obstructing the tubular lamina, especially in the distal tubule resembling a similar mechanism as myoglobin casts formation seen in rhabdomyolysis [1]. In Nayak et al.’s study, they were able to detect BCN in 44.8% of all the postmortem renal biopsy specimens and in 72.1% of the patients with acute on top of chronic liver failure [37]. These bile casts are formed by sloughed tubular epithelial cells. The Hall (or Fouchet) histochemical stain highlighting green to yellow casts or periodic-acid Schiff (PAS) stain showing red to dark red colored casts are used to confirm the presence of bilirubin [1,3]. The kidney may also show variable degrees of acute tubular injury, such as vacuolization of tubular cells and tubular necrosis. Contraindications in obtaining a kidney biopsy in liver disease and coagulopathic patients, coupled with difficulty securing distal nephron segments with conventional biopsy methods, have significant limitations in diagnosing this condition [1,3,10,30]. It is unclear whether bilirubin is truly nephrotoxic, but there is an increased likelihood that a patient may develop bile casts with prolonged exposure to increased bilirubin levels (i.e., bilirubin >20 mg/dL) [10,43,44]. Several case reports have documented bilirubin and creatinine levels in patients with BCN during their disease and most of the case reports detail that kidney function is noted to deteriorate as bilirubin concentration increases [2,5,8,9,18,19,32,34,38,42]. The majority of the studies show a parallel increase in creatinine with bilirubin.

Biomarkers

AKI is associated with high morbidity and mortality and can occur in patients with severe liver disease. Proximal tubule cells are sensitive to hypoxic injury, leading to a release of proteins into the urine. Several promising urinary biomarkers may be used to evaluate AKI. The most studied is neutrophil gelatinase-associated lipocalin (NGAL), a 25 kDa iron-transporting protein excreted in nephrotoxic or ischemic kidney injury. Urinary(u) NGAL levels in CN were shown to be suitable to monitor tubular epithelial damage and therapeutic effects under experimental conditions [1,45]. The diagnostic odds ratio (DOR) and sample size-weighted area under the curve for the receiver-operating characteristic (AUC-ROC) for NGAL to predict AKI were 18.6 (95% CI: 9.0-38.1)/0.815 (95% CI: 0.732-0.892) in a meta-analysis study of 19 studies with AKI [46]. Another marker, interleukin 18 (IL-18), is a known factor that induces interferon-γ and is closely related to the IL-1 cytokine family. It is found in dendritic cells, macrophages, and epithelial cells, which generate Th1 response, activate natural killer (NK) and cytotoxic T cells, and aid in proliferating T cells [47]. A marker known as kidney injury molecule (KIM-1), also known as T cell Ig and mucin domain 1 (TIM-1), is a phosphatidylserine receptor that aids in phagocytosis of apoptotic bodies and oxidized lipids, especially in chronic kidney injury. It interacts with p85 and downmodulates PI3K-dependent nuclear factor-κB [48]. A novel biomarker is the human liver-type fatty acid-binding protein (hL-FABP) which binds long-chain fatty acids and plays a role in fatty acid metabolism, thus being renoprotective as it promotes lipid excretion peroxidation products [49]. Animal studies have been conducted to ascertain the sensitivity of potential biomarkers; however, they have not yet been included for laboratory testing and are used exclusively for research [1]. A summary of the possible markers for kidney injury is seen in Table 2.

**Table 2:** Potential markers for kidney tubular injury. NGAL: neutrophil gelatinase-associated lipocalin; CBDL: common bile duct ligation; IL-18: interleukin-18; KIM-1: kidney injury molecule-1; L-FABP: human liver-type fatty acid-binding protein; mRNA: messenger RNA; kDa: kilodalton

Tubular injury biomarkers	Source	Description	Rationale	Studies in BCN
NGAL	Urine serum	25-kDa protein bound to gelatinase in the specific granule of neutrophil	Expression upregulated in kidney proximal tubule cells and urine following ischemic-induced renal injury	Increased urinary expression in CBDL mouse model, increased mRNA expression CBDL mouse model
IL-18	Urine serum	Interleukin 18 is a 24-kDa protein	Expressed in distal tubules; strong immunoreactivity in proximal tubules with transplant rejection; upregulated in ischemic injury	None
KIM-1	Urine	Type-1 cell membrane glycoprotein	Upregulated in dedifferentiated proximal tubule epithelial cells following injury	Increased mRNA expression in CBDL mouse model
L-FABP	Urine	14-kDa protein found in the cytoplasm of human renal proximal tubules	Expressed in proximal tubule epithelial cells	None

Treatment strategies and outcomes

The definitive diagnosis and subsequent treatment of CN are challenging, especially considering that a few case reports are diagnosed postmortem through autopsy, and adequate treatment was not provided. Treatment of CN is primarily based on treating the underlying cause of hyperbilirubinemia to prevent kidney injury [4]. In the case of biliary stones or tumors, endoscopic retrograde cholangiopancreatography, stent placement, and tumor resection may be done to relieve the obstruction. Extracorporeal therapies such as hemodialysis and plasma exchange are the other treatment options when CN is diagnosed; however, the number of sessions varies and the outcome [4]. Patients are reported to have clinical improvement and even complete kidney injury reversal evidenced by normalization of creatinine and reduction of bilirubin levels [14,16,19,20,22,39,42]. The time for recovery observed varied per study. Both Flores et al. and El Khoury et al. describe a case of CN due to anabolic steroid use successfully treated with several sessions of plasma exchange [26,34]. We report a detailed list of the therapies and treatment outcomes in Table 1.

Extracorporeal therapies are possible treatment options in patients with CN and are divided into biologic and nonbiologic. Biologic therapies use living liver cells, while nonbiologic therapies use artificial membranes and adsorbents (i.e., plasmapheresis and albumin dialysis). These therapies can reduce inflammatory cytokines and bilirubin levels. Plasmapheresis is when plasma is separated from the blood, filtered, and returned to the patient. This therapy aids in the removal of excess toxins and bilirubin and replenishes albumin, coagulation factors, and hepatic regenerative stimulating substances to improve the symptoms of the patient [4].

On the other hand, hemodialysis is a complicated process involving blood filtration and regulating fluid balance. The case report by Sens et al. [27] utilizes a molecular adsorbent recycling system (MARS) as a treatment before the patient receives simultaneous liver and kidney transplants. MARS is a non-biological extracorporeal therapy that uses an albumin-enriched dialysate to selectively remove the albumin-bound toxins from the blood using two separate dialysis circuits [4,50]. Steroids, cholestyramine, ursodeoxycholic acid, and lactulose have minimal benefit in treating CN patients [8]. Interestingly, norursodeoxycholic acid has been proven to alleviate CN in the experimental scenario of CBDL mice. This study highlights the importance of bile acids with their hydrophilic nature, which aids in kidney function improvement and could be a potential medical treatment for patients with CN [11].

Future implications

There are no systematic guidelines for diagnosing, treating, and managing a patient presenting with CN. CN is a diagnostic dilemma, and more reasonable diagnostic alternatives are crucial in approaching suspecting patients with CN. The transjugular approach for kidney biopsy may be an effective option considering the associated coagulopathic risks with traditional kidney biopsy in liver cirrhosis patients [8]. However, studies should center on the non-invasive options for diagnosis, evaluating patients with absolute contraindications to invasive procedures. Treatment methods with albumin dialysis can help detoxify the albumin-bound compounds such as bilirubin, bile acids, and other hepatotoxins [4]. Prospective studies should be conducted to evaluate clinically meaningful treatment options despite most studies reporting clinical improvement and kidney function return with hemodialysis or plasma exchange. It is also sensible to further inquire about the newer extracorporeal therapies such as MARS, coupled plasma filtration adsorption (CPFA), and plasma filtration adsorption dialysis [8]. With the advancements in identifying biomarkers, specific urinary biomarkers such as NGAL can facilitate differential diagnosis along with the conventional techniques as markers of renal excretory function (serum creatinine, cystatin C), urine microscopy, and renal ultrasound [1].

## Conclusions

CN is an uncommon diagnosis but a common finding in patients with liver disease. Suspicion and meaningful consideration should be given to BCN in non-respondents to HRS treatment. The kidney biopsy is an accurate diagnostic, and the transjugular approach can be a better alternative to traditional biopsy to expedite the diagnosis by simultaneously obtaining liver and kidney biopsies, also lessening the bleeding risk in high-risk patients. This review is crucial in suggesting the various mechanisms, diagnostic techniques, and treatment approaches to BCN.
